# Naturalizing Sense of Agency with a Hierarchical Event-Control Approach

**DOI:** 10.1371/journal.pone.0092431

**Published:** 2014-03-18

**Authors:** Devpriya Kumar, Narayanan Srinivasan

**Affiliations:** Centre of Behavioural and Cognitive Sciences, University of Allahabad, Allahabad, India; University G. d'Annunzio, Italy

## Abstract

Unraveling the mechanisms underlying self and agency has been a difficult scientific problem. We argue for an event-control approach for naturalizing the sense of agency by focusing on the role of perception-action regularities present at different hierarchical levels and contributing to the sense of self as an agent. The amount of control at different levels of the control hierarchy determines the sense of agency. The current study investigates this approach in a set of two experiments using a scenario containing multiple agents sharing a common goal where one of the agents is partially controlled by the participant. The participant competed with other agents for achieving the goal and subsequently answered questions on identification (which agent was controlled by the participant), the degree to which they are confident about their identification (sense of identification) and the degree to which the participant believed he/she had control over his/her actions (sense of authorship). Results indicate a hierarchical relationship between goal-level control (higher level) and perceptual-motor control (lower level) for sense of agency. Sense of identification ratings increased with perceptual-motor control when the goal was not completed but did not vary with perceptual-motor control when the goal was completed. Sense of authorship showed a similar interaction effect only in experiment 2 that had only one competing agent unlike the larger number of competing agents in experiment 1. The effect of hierarchical control can also be seen in the misidentification pattern and misidentification was greater with the agent affording greater control. Results from the two studies support the event-control approach in understanding sense of agency as grounded in control. The study also offers a novel paradigm for empirically studying sense of agency and self.

## Introduction

Most significant events in our lives involve the feeling of volition: that those events are to some extent generated by our ‘self’ [1]. The folk psychological notion of self is simply whatever ‘I’ subjectively experience as myself [2]. But, the processes underlying the subjective experience of the self are hidden from us making it one of the least understood mysteries of the human mind. In the current study, we try to tease apart and empirically capture aspects of self, related to sense of agency.

Many theories have been proposed that try to unravel the mystery behind this sense of self [3]. While the traditional notion of self conceptualizes it as the center of all our experiences, some have argued that a volitional self that causes actions might be illusory in nature [4]–[7]. Metzinger [2] in his ‘self model theory of subjectivity’ claims “No such things as selves exist in the world. For all scientific and philosophical purposes, the notion of a self – as a theoretical entity – can be safely eliminated.” (see [2] pp. 563). He does not deny the experience of self, but rather contends that all the properties that we attribute to ‘experiential self’ are the dynamic content of an integrated process called ‘the phenomenal self model’. Such ideas seem to negate folk notions and traditional viewpoints, which acknowledges the ‘self’ as the causal center of all our experiences.

Although the concept of ‘self’ has multiple dimensions, the aspect of self that we focus upon is the experience of being the agent of an action, i.e., a sense of agency (SoA). Even if an individual’s experience of ‘self’ is illusory or epiphenomenal), it must be closely tied to SoA [8] because this reflects a person’s experience while interacting with (and possibly controlling) the environment. Note that the concept of SoA is not unitary; it has been divided into different aspects like implicit and explicit self, sense of ownership, sense of authorship (the degree to which participants believe they have control over their actions) and sense of identification [8]–[11]. In this study, we focus on the sense of identification and sense of authorship.

A possible way to understand an epistemic entity like the ‘self’, without the need for explicit definition, is by ‘naturalizing’ it. Naturalizing an epistemic entity like ‘self’ would enable us to indirectly assess different aspects of ‘self’ on the basis of its behavioral manifestation. In the current study, we attempt to naturalize SoA in terms of control of perception-action events exhibited by the organism situated in a particular environment [12].

One theoretical framework linking subjective experience of self to perception-action events is the *event control approach* [13], [14]. The approach uses the notion of ‘control’ exercised by an individual in the form of regularity in natural order of the organism’s interaction with the environment [13]. In different contexts, control can be described as the ability to influence, direct or constrain the activity and behavior of people, objects or events. This ability manifests itself in some sort of action that is used to influence aspects of environment, and consequentially what we perceive [13], [15]. All such actions by an organism can be distinguished in terms of the spatiotemporal gap between the action and its perceived effect: Events where the action and its effect lie close to each other in space-time are called proximal events, while those that lie farther away in space-time are called distal events. For any given action, multiple effects may ensue, which entails a multitude of proximal and distal perception-action linkages organized in different hierarchical loops. Every loop maps on to the environment and can be evaluated in terms of the control over the perceived effect. According to the event-control approach, the distal-event system (perception and action planning) constrains the event towards which the effector-control systems (actions) are directed. ‘Self’ in this approach can be mapped on to these synergistically linked control loops, which are nested in a hierarchical control system. At any given moment the sense of self in an organism is attached to the control loop relevant for a current goal (among multiple goals that are being pursued in parallel) that affords the organism the ability to pre-specify, monitor and produce distal effects (i.e., ability to exercise control).

Minimally, a control system consists of a controller/agent, a controlled system and a target of control. To exercise control, an agent must be able to accomplish the following: (i) act upon the system, (ii) perceive the resulting impact on system, and (iii) compare this impact with an expected goal [16]. In the case when an action results in the expected perceptual outcome, the agent is said to be in control of the event. When an action does not result in the expected outcome, the event is experienced as *not* being under the control of agent.

Multiple theoretical approaches like perceptual control theory [17], theory of event coding [12], and the free energy principle [18] have emphasized and discussed the role of control in explaining perception, cognition, and action. Perceptual control theory argues that the notion of control is central in explaining how we interact with our environment [15], [17]. They suggest that the aspect of an action controlled by the agent is its perceived outcome rather than the action itself. This control is achieved by matching the actual perceptual outcome with the expected perceptual consequence, which can be evaluated at different levels. The theory of event coding [19] argues that control is achieved in two stages; firstly, at the action selection stage and secondly, when the action outcome is evaluated. Both the theory of event coding and perceptual control theory suggest that exhibited control influences the nature of perceptual input and action planning for any particular event and the interplay between perceptual events and action events is mediated by the exhibited control. The match between the sensory prediction and the actual outcome also forms the basis of a family of models explaining the mechanism behind sense of agency [20]. Such comparator models differ in terms of the type of comparisons made (e.g. the two step comparator model [21]), or in terms of the nature of control mechanism (feed-back, e.g., [22], versus feed-forward, e.g., [23]).

Several studies have indicated that the manipulation of control influences participant’s subjective experience and executive processes [15], [24]–[26]. Moore et al [25] manipulated the statistical contingency between a key press and occurrence of a subsequent tone and found a subjective expansion in perceived elapsed time between the key press and the tone with increase in control (higher contingency). Studies on the perception of time between an action and its consequent effect indicate a close link between the experienced control and the SoA [27]. Disrupting the activity in pre-motor cortex using TMS decreased the experience of control and resulted in depletion of SoA [28]. Desantis et al. [29] have shown that when people perceive themselves as the cause of their action (similar to subjects experiencing control) their SoA increases. One intriguing study [30] asked participants to control a virtual boat in a noisy environment and reach a goal point. The dependent measure was subject’s judgment of control. They found that when the environment noise led participants closer to the goal, they reported greater control suggesting that the participant achieving the intended goal is enough to produce a subjective feeling of control.

In majority of the studies, [24]–[25], [30] control has been varied along a single dimension. This is a simplistic view of control for a system containing feedback loops at multiple levels. In a complex environment an organism may exercise control along various dimensions and at multiple levels. Even for something as routine as driving a car, a person has to maintain cognizance of the destination and also has to control the vehicle so as to not damage it, follow the traffic rules, avoid other cars on the road, just to name a few. According to event control approach, emergence of ‘self’ depends on the level at which optimal or best control is achieved, in a given scenario involving the organism and the environment. However, little experimental evidence exists in support of a multi-level hierarchical control framework to explain SoA. To understand the role of control in a more ecological setting, we need a paradigm that considers that control over an event exists at multiple hierarchical levels. In addition, earlier studies have not made direct queries regarding a participant’s subjective experience of identification of one’s ‘self’ as a causal agent in the context of other potential agents.

We have developed such a paradigm that can investigate the link between event-control and the subjective sense of self [13] and which fits well with recent theoretical approaches linking action and perception [12]. We performed two experiments in an attempt to naturalize self, using a novel multi-agent paradigm that explores the link between the sense of agency and control (varying at multiple levels along a distal-proximal hierarchy). The paradigm consists of a scenario with multiple agents moving around on a computer screen, in which the participant has to determine which agent he/she controls by interacting with the environment and competing with other agents in an attempt to accomplish a shared goal. Participants also provided confidence ratings regarding their sense of identification with a particular agent as well as the sense of authorship for every trial. Both are important aspects of the SoA. The first experiment investigated the linkage between correctly identifying which agent is controlled and the control hierarchy. The second experiment further studied the relation between exercised control and sense of agency by focusing on misidentifications of identity as a function of the control hierarchy.

## Experiment 1

According to the event-control framework [13], identification and characterization of ‘self’ as an agent is dependent on the distal event regularity shared with the environment and the agent’s perception-action interactions with the environment. In our opinion, this ability to discriminate between self-generated actions and the actions of others forms the core of what we know as ‘self’. When we correctly identify with an agent in the world, the degree of identifiability should depend upon our control over the distal-event regularity. The event-control approach would predict that when there are multiple distal event loops, the degree of exercised control of distal-event systems would hierarchically determine the sense of self [13], [14]. In the first experiment, we empirically verified these predictions using a novel paradigm.

The paradigm consisted of a multi-agent game like scenario where participants control one of the several agents (in this case a yellow circle we call a ‘wolf’) using a joystick. The goal is to catch a pre-specified agent (his/her goal, a blue luminance-matched disc we call a ‘sheep’). This needs to be accomplished while competing with other perceptually similar, computer-controlled wolves, which may or may not have the common goal (to catch the sheep). The two variables of interest were control at the goal-level and the control at the perceptual-motor interaction level. The control at goal-level was operationalized in terms of the outcome of a given trial, i.e., whether or not the participant is successfully able to catch the sheep. The control at perceptual-motor level was manipulated in terms of the amount of control the subject can exercise via a joystick. The first higher level of control (goal-level control) is more spatio-temporally distal compared to the more proximal second level of control (perceptual-motor control). The purpose of the experiment was to study the effect of joystick (perceptual-motor) control on identification of the user-controlled wolf, and how this identification depends on the achievement of the higher-level goal of catching the sheep.

The hierarchical nature of the two levels of control was investigated by studying the interaction between control at the goal level and control at the perceptual-motor level on the sense of agency. We predicted a significant two-way interaction effect with the different measures of agency as a function of goal completion and perceptual-motor control. We hypothesized that in situations in which the participant catches the sheep, the actual magnitude of perceptual-motor control over the ‘wolf’s movement using the joystick would not matter and that identifying the ‘self’ with the ‘wolf’ and associated confidence ratings would be similar across different amounts of perceptual-motor control at the proximal level.

If the various control loops are arranged in a hierarchical relationship, then the event-control approach would predict that sense of self would depend upon the highest level at which control is achieved. Minimally, this would mean that lower level control loops would play lesser role in determining the sense of self, when control is achieved at higher levels. Returning to the example of driving, an experienced driver for whom driving is fairly automatic would find ‘self’ attached to the successful achievement of the goal, i.e. arriving at the correct destination. In our study, this would mean that when control at the goal-level is achieved, (‘goal completed’), control at the perceptual-motor level (joystick control) would not play a role in determining SoA. In contrast, when control at the higher level is not achieved, lower levels might play a greater role. For an inexperienced driver, sense of self would get attached to the proximal level of control related to hand or leg movements in controlling the clutch, brake, and gear. In our study, this would mean that when control at the goal level is not achieved (‘goal not completed), control at the perceptual-motor level (joystick control) would influence the SoA.

### Method

#### Participants

Sixteen naive volunteers with normal or corrected-to-normal vision from University of Allahabad provided written consent and participated in the experiment. The study was approved by the Ethics Committee of the University of Allahabad.

#### Stimuli and Apparatus

**Figure 1:**
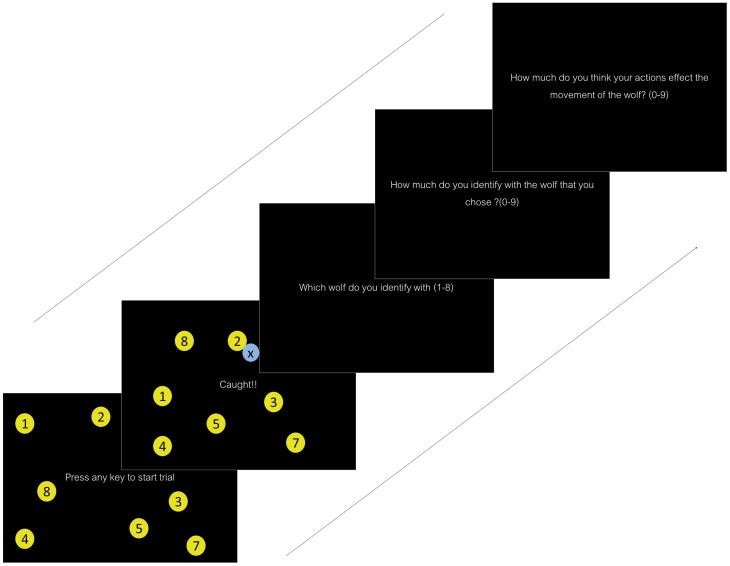
Structure of a single trial (figure not to scale) in Experiment 1. The stimuli consisted of multiple wolves (yellow circles) are wolves labeled with a number and a sheep represented by a blue circle labeled ‘x’.

Stimuli consisted of eight wolves and one sheep (see Figure 1) of which one wolf was partially controlled by the participant using a joystick. All the wolves were perceptually similar to each other (subtending 1.723° in size with identical shape and color). A unique number (from 1–8) was written at the center of the circle for every wolf. Participants were asked to use this number at the end of each trial to indicate which wolf they thought they controlled. The smaller sheep subtended 1.48° and the letter ‘X’ was written in the circle. Participants were seated at an approximate distance of 90 cm from the screen. The experiment was designed on MATLAB using Psychophysics Toolbox 3. Responses were recorded using Logitech Attack III joystick and a standard keyboard.

#### Design & Procedure

Participants’ task was to identify the wolf they controlled with the joystick and to catch the sheep while competing with the other wolves. They were later asked to respond to certain questions related to identifiability and control in a particular trial (see Figure 1). At the beginning of each trial a static display was presented to the subject, consisting of one sheep and eight wolves, initially placed at random positions on the screen. After three seconds, all the agents started moving, and the subject was able to influence movement of one of the wolves using joystick (the identity was initially not known to the subject).

Out of the eight wolves, four were chasers (including the user wolf) and four were non-chasers. The non-chasers moved randomly while the chasers followed a pre-specified heuristic to catch the sheep [31]. For all the chaser wolves (including the user wolf), a prediction was made regarding the path to catch the sheep using a modified heat sink algorithm (i.e., move in the direction of the sheep when the egocentric window between directions in which wolf is moving and direction in which sheep is moving becomes greater than 45 degrees). The computer wolves followed the path suggested by the algorithm. For the user wolf, a weighted average of direction suggested by joystick (*θ*_user_) and direction suggested by computer algorithm (*θ*_computer_) was taken. 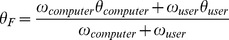



The independent variable being manipulated between trials was the amount of control afforded in a particular trial. This was done by changing the relative weights 
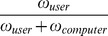
 at five equidistant levels, resulting in five levels of control ranging from no control to full control (i.e., at levels 0, ¼, ½, ¾, and 1). The movement of the sheep also followed a predefined algorithm, it moved in a direction that will take it away from the chaser wolves. The non-chaser wolves followed a random movement pattern and were programmed not to catch the sheep.

The participants were instructed that in every trial eight wolves would be presented on screen. Participants have partial control over one of the wolves and to complete the task successfully, they are required to identify the wolf they are controlling and try to catch the sheep before other wolves. They were informed that some of the wolves are chaser wolves (which will compete with them and try to catch the sheep) and some are non-chaser wolves (which will move around randomly and do not catch the sheep). Participant could identify the wolf only by controlling its movement on the screen. Each trial ended when one of the wolves caught the sheep.

At the end of each trial participants answered three questions about his/her subjective experience: (1) Which wolf were you controlling?, (2) How much do you identify with the wolf that you have chosen?, and (3) How much do you think your actions affect the movement of the wolf?, For the first question, participants indicated the numeric label of the identified wolf. For the second and third questions subjects used a nine point scale with 0 being ‘least’ and 8 being the ‘most’. The second question was a measure of the participant’s sense of identification with the agent. The third question measured the sense of authorship, i.e. the degree to which the subject believed he/she had control over his/her actions. For every trial, Subjects’ response for these three questions was recorded along with a measure of whether or not subjects are able to achieve the goal (catch the sheep). There were 75 trials in the experiment (15 trials per level of perceptual-motor control) with participants averaging 50–60 minutes to complete the experiment.

### Results and Discussion

In the ‘no-control’ condition there was no user wolf and all the wolves were controlled by computer. In these trials, ‘correct identification’ and ‘goal completion’ were not defined. Hence, we dropped the zero control condition from further analysis. To check perceptual-motor control and goal completion was independent, we performed a one-way repeated measures ANOVA with perceptual-motor control as the independent variable on proportion of trials in which participants completed the goal. There was no significant effect, *F*(3, 45)  = 0.78, *p* = 0.51, suggesting that ‘goal completion’ and ‘perceptual-motor control’ can be treated as independent of each other (mean win proportion was 0.22).

We performed two-way ANOVAs with perceptual-motor control (4 levels: 1/4th, 2/4th, 3/4th, and full control) and goal completion (goal completed and goal not completed) as independent variables and the percentage of maximum possible response (POMP score) for identification and authorship rating as the dependent measure. POMP score is used to combine the number of responses and rating for each response to make meaningful interpretation regarding data, especially when the number of items per condition is not the same. This measure takes into account any kind of biasing caused by unequal item responses (see [32] pp. 156). POMP score is a normalized score obtained by dividing the sum of all the response ratings for a particular goal completion condition (Σf_i_*x_i_) by the product of number of trials in the goal condition and maximum possible rating. To handle the missing data (7 out of 128 possible cells across all conditions and participants), we used the multiple imputation technique (Amelia II [33]).

The main effect of control was significant for POMP score of authorship rating, *F* (3, 45)  = 6.04, *p*<.01 but not for identification rating, *F* (3, 45)  = 1.82, *p* = .15. Post-hoc analysis (Tukey corrected) suggests that POMP score increases with increase in perceptual-motor control, authorship rating (1/4^th^ Control< Full Control, *t*(45)  = 5.04, *p*<.01, 1/4^th^ Control<3/4^th^ Control, *t*(45)  = 3.58, *p*<.05, 2/4^th^ Control<Full Control, *t*(45)  = 3.4, *p* = .08). The main effect of goal completion on POMP score was also significant for both identification rating, *F*(1, 15)  = 28.66, *p*<.01, as well as for authorship rating *F*(1, 15)  = 36.32, *p*<.01, with greater POMP score for trials in which the goal was completed compared to trials in which goal was not completed.

**Figure 2:**
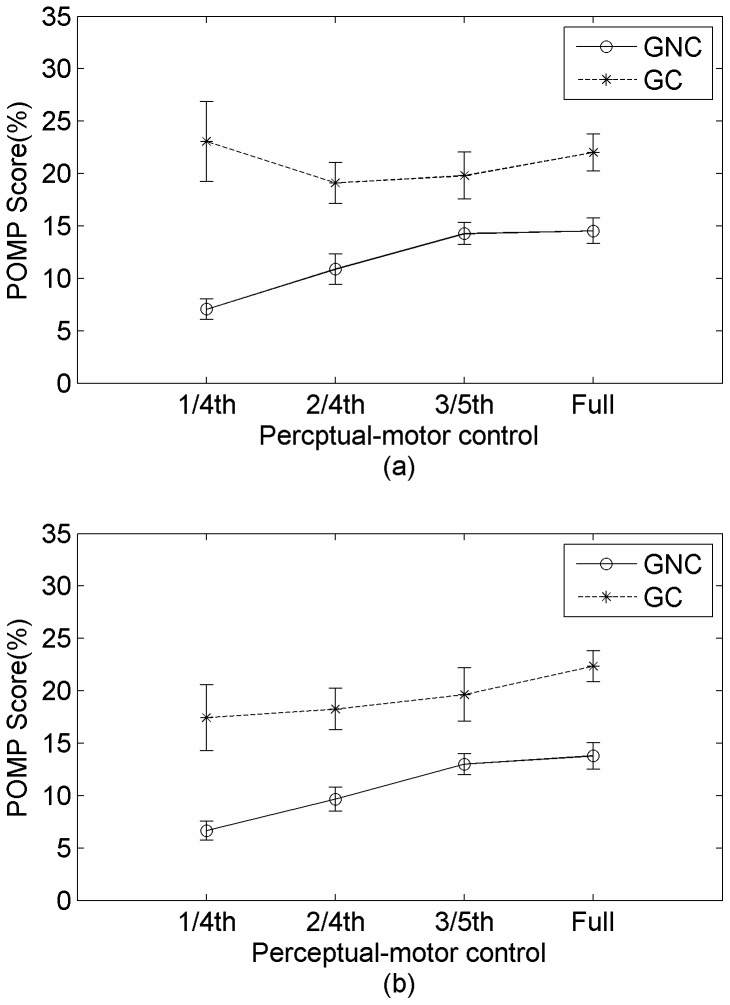
Percentage of maximum possible scores for (a) identification ratings and (b) authorship ratings in correctly identified trials as a function of goal completion (GC- Goal Completed, GNC- Goal not completed) and perceptual-motor control in Experiment 1.

We were mainly interested in how the goal completion and the perceptual motor control interact with each other, specifically the difference in the POMP score between each control condition when goal was completed and when the goal was not completed. The interaction between goal completion and control was significant, for the identification rating, *F*(3, 45)  = 2.83, *p*<.05, but not for authorship rating, *F*(3, 45)  = 0.43, *p*<.69 (see Figure 2). Post-hoc analysis (Tukey corrected) for identification rating showed that when subjects did not complete the goal, there was an increase in POMP score with increase in control (identification rating for 1/4th control<3/4th control, *t*(45)  = 3.76, *p*<.05, 1/4th control<Full control, *t*(45)  = 3.9, *p*<.05). However, when subjects completed the goal, post-hoc analysis showed no significant difference between the identification ratings between the four control conditions, suggesting that POMP score is not influenced by control (all *p*s>.46).

To ensure that the effect of goal completion and control on identification and authorship ratings was not due to the duration of a particular trial, we conducted a two-way repeated measures ANOVA between perceptual-motor control and goal completion on trial duration. The effect of perceptual-motor control, *F*(3,42)  = 0.81, *p* = .49, and goal completion, *F*(3,42)  = 1.15, *p* = .30 was not significant. Also, the interaction between perceptual-motor control and goal completion was not significant, *F*(3, 42)  = 0.67, *p* = .57.

Results support the hypothesis that levels of perceptual-motor control and goal completion hierarchically influences the sense of agency in agreement with the predictions made by the event-control approach. The rating for sense of identification increased with perceptual-motor control when goal-level control was not present (when goal is not completed). When control was exercised at the higher level, (when the goal of catching the sheep is completed) the lower level perceptual-motor control did not influence identification rating. In terms of hierarchy, the lower level control loop (associated with the movement of the joystick and actual movement of the wolf) played a role in the emergence of self, only when control was not achieved at the higher level (the goal of catching the sheep), indicating close links between exercised control and sense of self. These results are similar to that by Kumar and Srinivasan [11], where authors showed a hierarchy of control using intentional binding as the measure of agency.

It is to be noted that the notion of control in such a scenario is neither completely internalized (felt control) nor completely externalized (affordance of control). Rather it is a combination of our high-level goal and planning at a distal spatio-temporal level and proximal perception-action control or even the effector control system. The regularities linked to the emergence of self as explicated in this novel perception-action paradigm may provide a robust method to naturalize and study self in an empirical manner. The hierarchical nature of these control loops that influences the experience of self might provide a way to model the SoA.

### Experiment 2

Experiment 1 demonstrated how different hierarchical levels of control influence the participant’s sense of identification. Any framework trying to explain the ‘self’ in a multi-agent environment should also be able to account for situations in which the participants identify with an agent they did not control (false attribution). Focusing on how misidentification occurs would help in understanding the mechanisms underlying our sense of agency [21]. In Experiment 2, we investigated the misidentification errors as a function of event-control hierarchy.

In a multi-agent environment, participants should typically identify with the agent that affords greatest amount of control. When there are multiple agents in a low control situation, there is a possibility that some other agent will by chance alone provide a better match with the predicted outcome of our actions resulting in a greater sense of ‘felt control’ and hence more misidentifications as the agent being controlled.

In Experiment 2, participants could make three kinds of attributions during identification with a particular agent. First, participants could identify correctly the agent (‘wolf’) they were controlling (the user-controlled chaser). Second, participants could misidentify with a computer-controlled agent that was also chasing the sheep (the computer-controlled chaser). Third, participants could misidentify with an agent controlled by computer but moving randomly (the computer non-chaser). In terms of control, both the computer-controlled and the user-controlled chaser afforded goal completion (catching the sheep). The possibility of affording perceptual-motor control (the match between joystick movement and movement of wolf on the screen) thus existed for all three types of wolves.

The event-control approach suggests that goal-level control and perceptual-motor control would interact hierarchically with each other in determining the sense of ‘self’ [13], [14]. Hence, hierarchical relationship would be predicted when participants correctly identify with the wolf they control. For correctly identified trials, our predictions were similar to those in the first experiment: When participants do not complete the goal (computer catches the sheep), POMP score for identification would increase with increase in perceptual-motor control. When participants complete the goal (user catches the sheep) there would be no change in POMP score along with control, for identification. The lack of interaction between the two levels of control in experiment 1 for sense of authorship might have been due to the fact that there were more wolves with the same goal as that of the user-controlled wolf making it dependent only on perceptual-motor control. We reduced the number of computer-controlled chaser wolves in experiment 2 (to one) to see whether this would result in an interaction for sense for authorship.

The computer-controlled chaser and computer non-chaser were physically indistinguishable from each other. The only thing that differentiated them was the fact that the chaser wolf was more likely to give rise to the illusion of the goal-level control (because it tries to catch the sheep) whereas a non-chaser wolf affords far less of such a possibility. Furthermore, when participants completed the goal, there might be little difference between the non-chaser wolf and chaser wolf, and people might be likely to misattribute as being either of them. But when computer-controlled wolf caught the sheep, then according to event-control approach people should identify more with the computer-controlled chaser compared to computer non-chaser. Hence we hypothesized that when participants caught the sheep (and completed the goal), POMP score for identification and authorship would be the same for both identification as computer-controlled chaser and non-chaser. However, when computer-controlled chaser caught the sheep, the POMP score for identification and authorship would be higher for identification for the computer-controlled chaser compared to computer-controlled non-chaser.

### Methods

#### Participants

Twenty two naive volunteers with normal or corrected-to-normal vision from university of Allahabad provided written consent and participated in the experiment. The study was approved by the Ethics Committee of the University of Allahabad.

#### Design and Stimuli

The apparatus was the same as that used in the first experiment. Similar to the first experiment, the current experiment consisted of a sheep, a user-controlled wolf and computer-controlled wolf. However, instead of eight, the total number of wolves in the second experiment was four (one user controlled chaser, one computer chaser and two non-chasers). In the second experiment, the independent variable perceptual-motor control (control offered by joystick) was manipulated at three levels (1/3^rd^, 2/3^rd^, and Full) compared to the five levels of manipulation in the first experiment. This was done to make the overall design simpler.

We also introduced two other factors, strength of competitor and congruency, to counterbalance the goal level control and perceptual-motor control across the design and make them relatively orthogonal. Strength of competitor was manipulated at two levels by changing the threshold angle *θ* that determines how accurately a chaser wolf follows the sheep [31]. With smaller *θ* the computer agent becomes a better chaser. In the first experiment, this angle was fixed. Here, we manipulated *θ* at two levels (0° and 75°), with computer wolf being a better chaser (lesser chance of participants catching the target sheep decreasing the goal-level control for them) when *θ* is ‘0°’ and computer wolf being a worse chaser (greater chance for participants to catch the sheep) when *θ* is ‘75°’. For the factor congruency, we manipulated whether the direction in which joystick was moved and the user sheep moved was same (congruent condition) or the user wolf moved at an angle of 30° to either left or right of the direction of joystick movement. We counterbalanced both the factors of strength of competitor as well as congruency across the three conditions of control. ‘Strength of competitor’ and ‘Congruency’, were not part of our analysis. Also, to minimize the effect of trial duration, we fixed the maximum trial duration to 60 seconds.

#### Procedure

The participants’ task was same as in Experiment 1, to identify the wolf being controlled, catch the sheep and then provide confidence ratings. They were instructed that there was a one-minute limit to each trial, after which the trial ended with a message ‘Time Up’ displayed on the screen. These trials in which neither the user wolf nor the computer-controlled chaser wolf caught the sheep were not included in further analysis. After the trial was completed, participants were given questions that were same as those in Experiment 1. There were 72 trials in the experiment with participants averaging 45 minutes to complete the experiment.

### Results and Discussion

#### Sense of Identification

**Figure 3:**
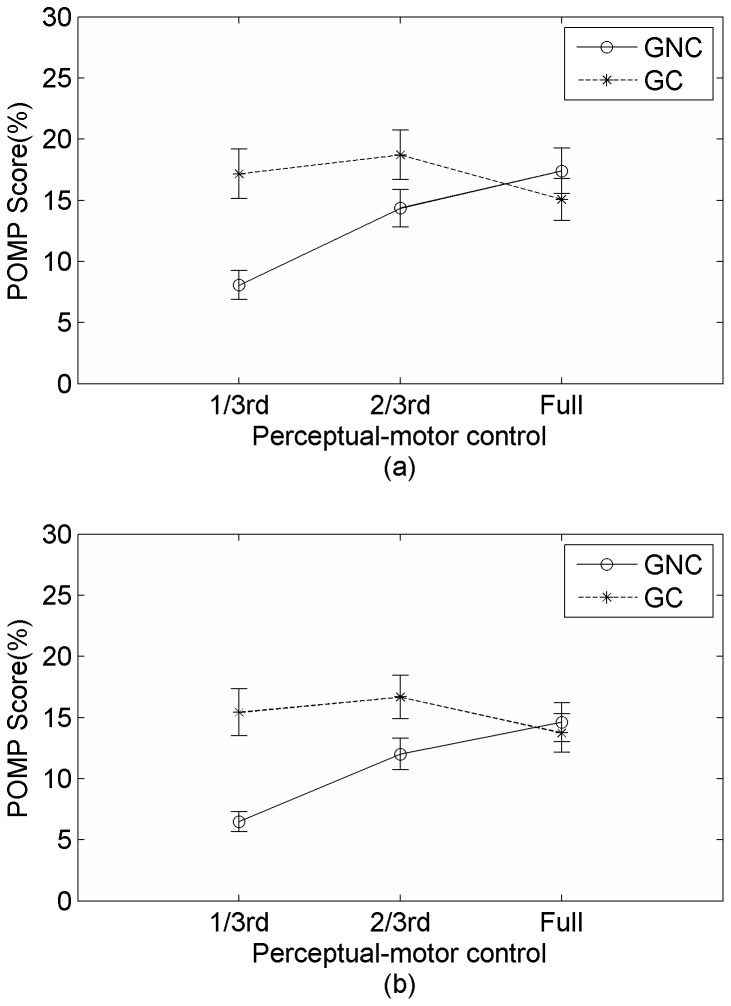
Percentage of maximum possible scores for (a) identification ratings and (b) authorship rating as a function of goal completion (GC-Goal Completed, GNC- Goal not completed) and perceptual-motor control when subjects identified with participant-controlled chaser in Experiment 2.

We performed a two-way ANOVA with perceptual-motor control (3 levels: 1/3^rd^, 2/3^rd^, full) and goal completion (2 levels: goal completed, goal not-completed) as independent variables with POMP scores for identification and authorship ratings. The analysis for identification ratings showed a main effect of perceptual-motor control, *F*(2, 42)  = 3.9, *p* <.05 (identification rating increased as a function of perceptual-motor control, 1/3rd control <2/3^rd^ control, *t*(42) = 3.75, *p*<.05, 1/3^rd^ control<2/4rd control, *t*(42)  = 3.47, *p*<.05) and a main effect of goal completion, *F*(1, 21)  = 9.17, *p* <.01 (with identification score being higher for ‘Goal Completed’ condition compared to ‘Goal Not Completed’ Condition). More importantly, similar to Experiment 1, the two-way interaction between perceptual-motor control and goal completion, *F*(2, 42)  = 8.77, *p*<.01, was significant. Post-hoc analysis (Tukey corrected) showed that when subjects identified correctly as the user-controlled chaser but did not complete the goal, the POMP score for identification increased with amount of control (1/3^rd^<2/3^rd^, *t*(21)  = 4.26, *p*<.05, 1/3^rd^< full, *t*(21)  = 6.32, *p*<.01, 2/3^rd^< full, *t*(21)  = 2.06, *p* = .32). When subjects correctly identified the user-controlled chaser and completed the goal, there was no difference between in identification scores across different perceptual motor control conditions (all *p*s> 0.2). The results are similar to those from Experiment 1 and support our hypothesis regarding hierarchical interaction between the two levels of control in determining sense of agency (see Figure 3).

#### Sense of Authorship

The analysis of POMP score for authorship confidence rating showed a main effect of perceptual-motor control, *F*(2, 42)  = 14.88, *p*<.01 (identification rating increased as a function of perceptual-motor control, 1/3rd control < 2/3^rd^ control, *t*(42)  = 3.67, *p*<.05, 1/3^rd^ control < full control, *t*(42)  = 3.49, *p*<.05), and goal completion, *F*(1, 21)  = 4.09, *p*<.05 (with identification score being higher for ‘Goal Completed’ condition compared to ‘Goal Not Completed’ Condition). More importantly, the two-way interaction between goal completion and perceptual-motor control, *F*(2, 42)  = 7.79, *p*<.01, was significant. Post-hoc analysis showed that when subjects correctly identified as the user controlled chaser but did not complete the goal, the POMP score for identification increased with amount of control (1/3rd<2/3^rd^, *t*(21)  = 4.23, *p*<.05, 1/3^rd^< full, *t*(21)  = 6.23, *p*<.01, 2/3^rd^< full, *t*(21)  = 2, *p* = .34). When subjects correctly identified the user-controlled chaser and completed the target, there was no difference between different control conditions (all *p*s>.25). This result is similar to that for identification and lends further support to the event-control approach. The results from both experiments with identification and authorship ratings support the idea that the sense of self and agency depends hierarchically on amount of control afforded at various levels (in this case at joystick level and at goal completion level).

#### Misattribution Analysis

**Figure 4:**
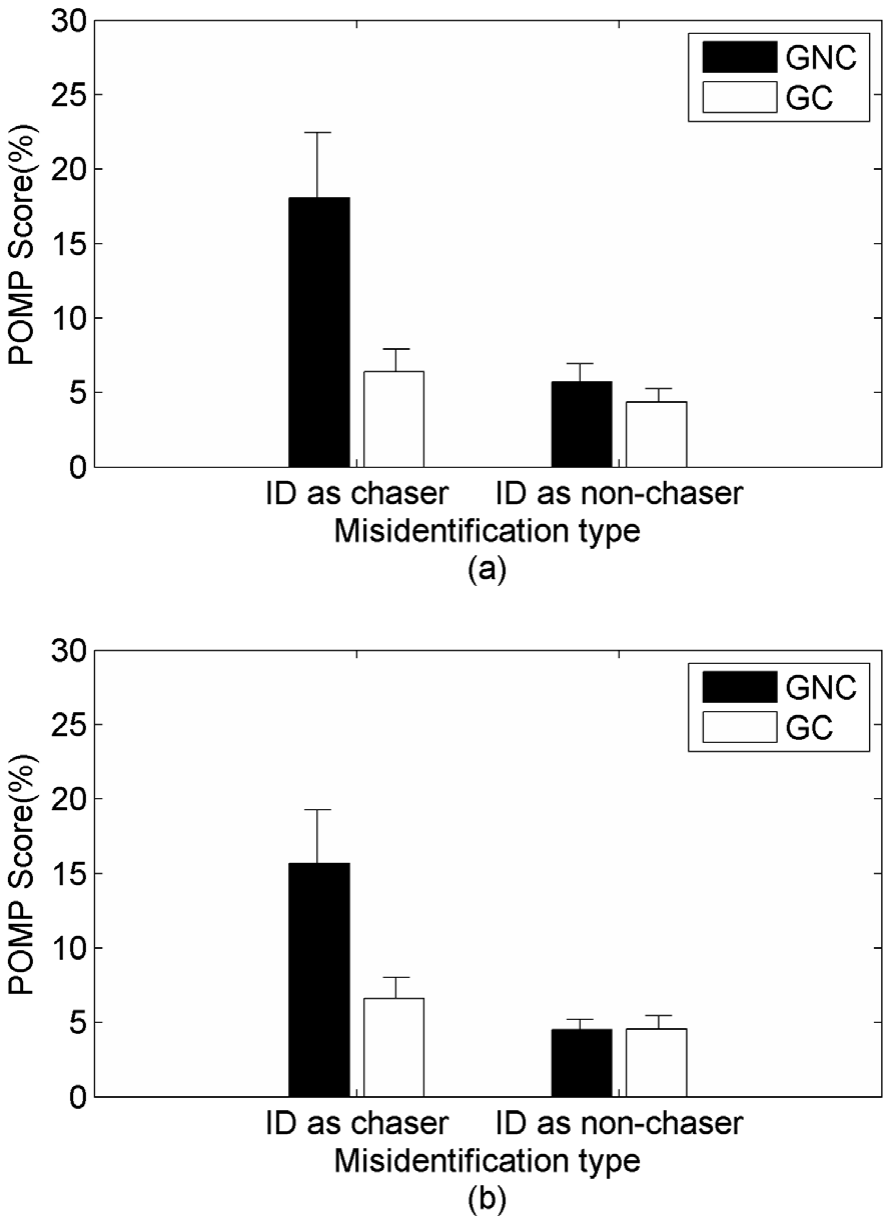
Percentage of maximum possible scores for (a) identification and (b) authorship ratings as a function of goal completion (GC-participant chaser completes the goal, GNC- computer chaser completes the goal) and misidentification type (Id as chaser- misidentification as computer chaser, Id as non-chaser- misidentification as computer non-chaser).

To test the hypothesis for misidentification patterns, we conducted two-way repeated measures ANOVA with misidentification type (computer-controlled chaser and computer non-chaser) and goal completion as independent variables with the POMP score for identification as well as authorship as dependent measures (see Figure 4). We found a significant main effect for both identification type, with a greater POMP score for identification as chaser (identification rating: *F* (1, 21) = 6.26, *p*<.05 and authorship rating: *F*(1, 21)  = 6.89, *p*<.01) as well as for goal completion, with a higher score for trials in which goal was not completed compared to trials in which goal was completed (identification rating: *F*(1, 21)  = 10.41, *p*<.01 and authorship rating: *F*(1, 21)  = 9.92, *p*<.01). The interaction between goal completion and misidentification type was significant for identification rating, *F*(1, 21)  = 6.89, *p*<.01 and for authorship rating, *F*(1, 21)  = 10.08, *p*<.01.

Post-hoc comparisons (corrected for homogeneity) showed that when the user wolf completed the goal, misidentification POMP scores for computer-controlled chaser and computer non-chaser were not significantly different (for identification rating: *t*(21)  = 0.59, *p* = .68 and for authorship rating: *t*(20)  = 0.01, *p* = .99). When the computer-controlled chaser completed the goal, misidentification score for computer-controlled chaser was significantly greater than that for computer non-chaser (for identification rating: *t*(20)  = 5.01, *p*<.01 and for authorship rating: *t*(20)  = 4.86, *p*<.01). The result supports our hypothesis that participants would misidentify more with the computer-controlled chaser compared to the computer non-chaser only when computer-controlled chaser completes the goal.

To summarize, we found a significant hierarchical interaction between perceptual-motor control and goal level control in determining sense of identification when participants identify with the user chaser. Also, we found evidence supporting event control approach when participants misidentify with another agent. Specifically we found that participants tend to misidentify more with the agent that affords greater control. This validates the event-control approach even under cases of misidentification.

## General Discussion

Unlike earlier approaches that have tried to address questions regarding the self and sense of agency, the event-control approach [13]–[14] takes into account the importance of control at different hierarchical levels in order to explain the emergence of the sense of ‘self’ and provides a basis from which to study this in a more empirical fashion. We used a novel paradigm involving the dynamic interaction between the organism and its environment consisting of perception-action cycles enabling us to empirically study the SoA through embodiment in a multi-agent setting. We naturalized SoA in terms of hierarchical control that our participants could exercise in this multi-agent situation.

Results from both the experiments show that the sense of identification depends closely on the incremental control that an individual can exercise. Accuracy and confidence in identification depends on the amount of control that can be exercised. The results indicate that the subjective sense of self (and agency) depends on the control (or constraints) of the action that we perform to produce desired changes in the environment. Such an environment may not be fully predictable and indeed is often noisy [34] due to changes in the environment as well as the presence of others agents sharing the same environment. These disturbances in the environment necessitate feedback and possibly predictive control on the part of the agent [16], [20]. This control exercised by the agent influences the agent’s SoA. In addition to the environmental noise, presence of other agents in the environment complicates the prediction of action outcomes and influences the participant’s perceived and actual control of the perception-action events. The fine-tuning of control provided by discriminating between which agent is controlled and which are not is central the development of self and agency.

An important aspect that forms the core of the event-control approach is that control is exercised at multiple levels in a hierarchical control system [13], [17], [20], [35]–[38]. Our results are consistent with hierarchical predictive control system frameworks that underlie perception, action, and consciousness (20,34,38–39). Both the experiments in the current study clearly show that the subjective sense of identification and authorship changes hierarchically with changes in control at various levels. The distal control loops lie (e.g., goal level control) higher in the hierarchy compared to lower levels (e.g., perceptual-motor control). Proximal control loops have an influence only when control at the higher levels is not achieved. Hommel [36] has argued for an offline ideomotor system that would include conscious goals at the higher level and online sensorimotor system at the lower level. Marken [37] has shown that the relationship between variables that are hierarchically related showed the emergence of a coordinated structure when one variable influences the consequence that is being controlled by the other variable indicating the importance of control hierarchy.

When people are given a self-identification task in a multi-agent setting in which identification is difficult, they might make incorrect identifications. This is apparent in volition related disruptions, in which participants either stop identifying with what they own, like in alien hand syndrome, or they start identifying with something that they do not own, like in rubber hand illusion [22]. Results from our second experiment show that in a noisy environment, people tend to misidentify themselves with non-user controlled agents who afford the appearance of more goal-level control. For example, people tend to identify themselves with the computer-controlled chaser more often when the computer-controlled chaser is the winner wolf (i.e., conforms to their goal-level control). The effect of goal-level control on agency seen for identification with the user-controlled chaser in both experiments 1 and 2 was also present when identified with computer-controlled chaser with whom the user-controlled chaser shared a common goal.

In addition to emphasizing the importance of hierarchy, our approach extends predictive control to regularities. The concept of predictability largely locates the ‘self’ inside the organism while the notion of event-control at different nested hierarchical levels (proximal or distal) encompasses both the organism and the environment and emphasizes the interaction between them [13]. Given that the environment of an agent contains other agents, we have developed a novel multi-agent scenario with shared goals (we often need to take another agent into account for correct prediction of environmental regularities). The presence of multiple agents and different levels of hierarchy enables us to go beyond studies looking at the effect of control on agency and perception-action integration [25], [30], [40].

The control of perception using a hierarchical event-control approach can also be linked to the free energy approach proposed by Friston and colleagues [18]. Free energy principle (FEP), suggests that the main task of an organism is to maintain its internal state in face of constantly changing environment. To perform this task, the system evaluates improbability of sensory data. According to this approach, the system tries to minimize this improbability or surprise by, optimizing predictions of the model to match the sensory information (‘Predictive coding’), or by selectively biasing certain sensory information that better confirms the predictive model (‘active inference’). FEP suggests that the sense of agency and sense of ‘mineness’ are implicit in the flow of information within a ‘hierarchical generative self model’ [41]. Our approach emphasizes the importance of control and the fluid aspect of sense of agency in a hierarchical control system that complements similar proposals based on the free energy approach [20], [41]–[42]. The additional emphasis on perception and action in the presence of other competing agents who share the same goal that we feel is critical to extend approaches like FEP.

The current study based on hierarchical control does not address the neural structures underlying the different hierarchical levels. Neural hierarchies have also been proposed in the context of cognitive control with the rostral regions of prefrontal cortex linked to more abstract control and the caudal regions of the prefrontal cortex linked to more concrete control [43]. In the context of our study, perhaps such an anatomical distinction could map on to the differences in the way in which the self is identified as a function of the level in the hierarchical event control approach.

Feinberg [39] has proposed a triadic neurohierarchical model of ‘self’, composing the interoself system, integrative self, and exterosensorimotor system with each of these systems containing multiple levels arranged in a nested hierarchical fashion. Our model is functionally similar to the model of Feinberg [39] in terms of the emphasis on hierarchy. However, we go beyond it to investigate and discuss how these functional event-control loops are often couplings between the organism and the environment, instead of focusing solely on the hierarchy of neural structures.

To conclude, we provide direct empirical evidence for the event-control approach using a novel paradigm consisting of multiple agents with shared goals, so as to understand how SoA depends upon control. The notion of hierarchical control is critical for the event control approach with specific levels in the control hierarchy, i.e., the highest level at which control is achieved, determining the sense of ‘self’. The nature of the sense of agency as evidenced by efficient control at a particular level would also have implications for other aspects of ‘self’. Further studies are needed to understand how different aspects of self and conscious experience are influenced by control.
